# Morphologic Heterogeneity in Carcinosarcoma of the Gallbladder: Report of a Rare Cases

**DOI:** 10.1155/2011/929654

**Published:** 2011-10-24

**Authors:** Vani Krishnamurthy, Sheela Devi C. Shivalingiah, Sunila Ravishankar, Gubbanna V. Manjunath

**Affiliations:** Department of Pathology, JSS Hospital, MG Road, Mysore, Karnataka 570004, India

## Abstract

Carcinosarcoma is a rare tumor composed of variable proportions of carcinomatous and sarcomatous elements and comprises less than one percent of all gallbladder malignancies. In most reported cases of carcinosarcoma of gallbladder, the epithelial component is adenocarcinoma. The mesenchymal component varies from homogenous sarcoma to more heterotopic elements like malignant bone, cartilage, and other mesenchymal tissues. We report a rare case of carcinosarcoma of the gallbladder in an 83-year-old male, with the carcinomatous component represented by undifferentiated carcinoma (spindle and giant cell type with osteoclastic giant cells) and the mesenchymal component seen as foci of chondrosarcoma.

## 1. Introduction

Carcinosarcoma, a rare tumor with grave prognosis, is characterized by malignant epithelial and mesenchymal elements. Occurrence of the tumor in many different organs including uterus, lung, esophagus, kidney and pancreas is well known [1]. However the gallbladder is a rare site with only 34 cases reported in the English literature to date [2]. We report a rare-case of carcinosarcoma in the gallbladder in an 83-year-old male. The carcinomatous component of the case is represented by undifferentiated carcinoma (spindle and giant cell type with osteoclastic giant cells), and the mesenchymal component is seen as foci of chondrosarcoma. 

## 2. Case Report

An eighty-three-year old male consulted the general surgeon for pain abdomen of fifteen-day duration. On clinical examination, he had tenderness in the right hypochondriac region. An ultrasound scan suggested empyema of the gallbladder with an abscess in left lobe of the liver. Considering the age of the patient and the associated conditions like essential hypertension and type 2 diabetes, laparoscopic cholecystectomy was planned. During laparoscopy, dense adhesions were noted around the gallbladder. The adhesions could not be released, and the abdomen was opened. The gallbladder was found to be necrotic and friable and was removed in piecemeal. The other abdominal organs were normal. The patient developed atrial fibrillations and died of cardiogenic shock on the tenth postoperative day before histopathological diagnosis was made. Hence no efforts were made to actively look for metastatic lesions. Consent for the autopsy was not given by the relatives.

Grossly, the specimen consisted of multiple grey white to grey brown friable fragments of tissues weighing approximately 300 grams.

**Figure 1:**
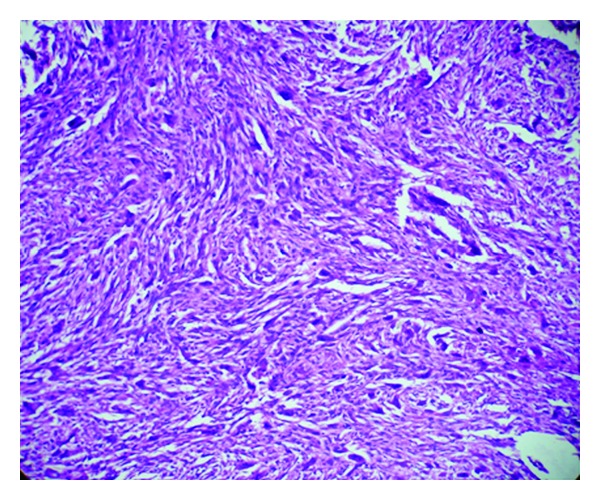
Spindle-shaped bizarre tumor cells along with pleomorphic tumor giant cells and osteoclastic giant cells (H&E, 100x).

**Figure 2:**
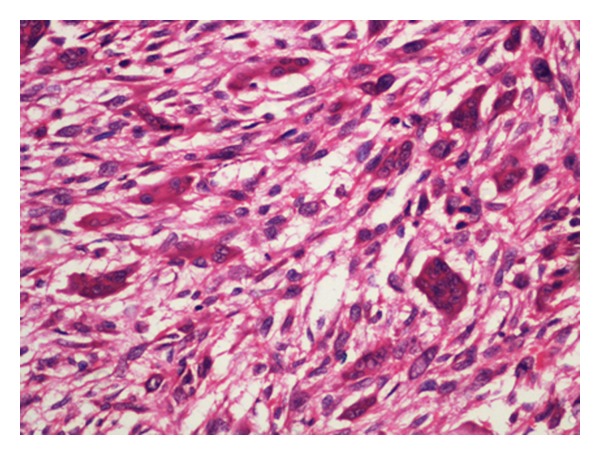
High power view showing bizarre spindle-shaped tumor cells along with osteoclastic giant cells (H&E, 400x).

**Figure 3:**
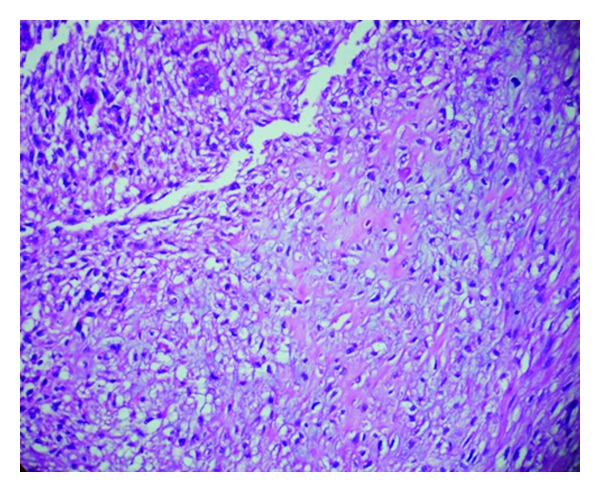
Focal chondrosarcomatous area amidst undifferentiated carcinoma (H&E, 400x).

Histology revealed fragments of gallbladder tissue displaying infiltrating tumor composed mainly of spindle-shaped pleomorphic cells with occasional bizarre giant cells (Figure 1). Dispersed osteoclastic giant cells were seen throughout the tumor tissue (Figure 2). Focal area showed chondrosarcoma (Figure 3). Infiltration of muscular layer was noted. However no normal gallbladder lining or lining with dysplastic features seen after studying multiple sections. Large areas of hemorrhage and necrosis were seen. Scattered inflammatory cells, congested capillaries, and dense fibrocollagenous tissues were also noted.

**Figure 4:**
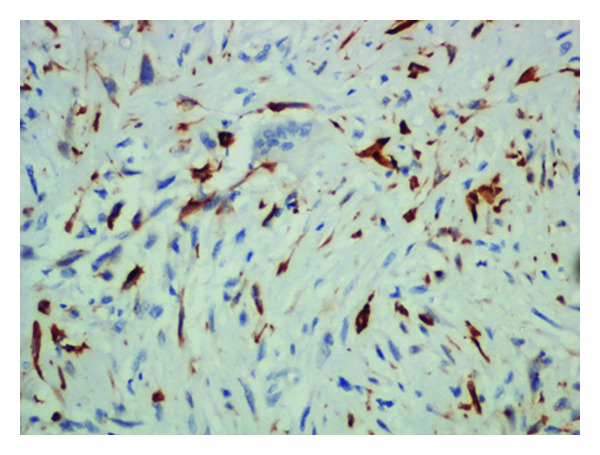
Spindle-shaped tumor cells showing cytoplasmic positivity for cytokeratin (IHC for cytokeratin, 200x).

On immunohistochemistry, majority of the spindle-shaped tumor cells and bizarre giant cells were positive for cytokeratin (Figure 4) with C-11, and 1 : 100 dilution (manufacturer—Biogenic) antibody was used. Osteoclastic giant cells were CD68 positive. 

A diagnosis of carcinosarcoma of the gallbladder was made.

## 3. Discussion

Carcinosarcoma of the gallbladder is a rare tumor with only 34 cases being reported in the English literature [2]. It is characterized by malignancy of both epithelial and mesenchymal components of the same tissue. Its diagnosis requires the presence and intermingling of both histological components [3]. The histogenesis is unclear. It is debatable whether it is due to concurrent transformation of epithelial and mesenchymal cell lines in the same organ or that the spindle cell component represents sarcomatous metaplasia in a poorly differentiated carcinoma [4].

The first case of carcinosarcoma of the gallbladder was reported by Landsteiner in 1907 in a museum specimen [1]. Most of the patients are females in their sixth or seventh decade and present with abdominal pain and right upper quadrant mass with or without jaundice [4]. In 74% of the cases, gall stones were also present. The tumor is usually a polypoidal mass filling the lumen that may be seen preoperatively on ultrasonography [4].

 In our case, the patient was an 83-year-old male who presented with abdominal pain of short duration. Ultrasonography suggested empyema of gallbladder with an abscess in the left lobe of the liver. There were no gallstones. Macroscopically, the specimen consisted of multiple fragments of friable tissue only.

 In most reported cases of gallbladder carcinosarcoma, the epithelial component is adenocarcinoma although a squamous cell carcinoma component may often be present. The mesenchymal component varies from homogenous sarcoma to more heterotopic elements such as malignant bone, cartilage, and other mesenchymal tissues [3]. 

Histologically in our case osteoclastic giant cells were the striking component amidst the undifferentiated carcinomatous areas with the mesenchymal component being focal chondrosarcoma. There were no foci of demonstrable adenocarcinoma or squamous cell carcinoma in the tumor.

Undifferentiated carcinoma of the gallbladder is an uncommon neoplasm and includes four morphologic variants, spindle and giant cell type, small cell type, lobular type, and osteoclast-like giant cell type [5]. In our case, the tumor seemed to be almost entirely composed of undifferentiated carcinoma spindle and giant cell type with osteoclastic giant cells. Extensive sampling and diligent search revealed focal minimal chondrosarcomatous element leading to a diagnosis of carcinosarcoma. However, none of the reported cases of undifferentiated carcinoma of the gallbladder have described heterologous elements such as osteoid, cartilaginous, or rhabdoid differentiated cells in the tumor.

The prognosis of carcinosarcoma is dismal with most patients dying within few months of diagnosis [4]. The invasive nature and aggressive malignant biology explains the limited number of resectable cases, though surgery remains the only curative management option [3]. Prognosis is poor following curative resection because of systemic metastasis to the liver and peritoneal dissemination [3]. In the present case, the patient died on the tenth postoperative day and within 25 days of presenting to the hospital.

## 4. Conclusion

Carcinosarcoma, a rare subset of gallbladder malignancy, usually has adenocarcinoma or squamous cell carcinoma as the epithelial component. Undifferentiated carcinoma, seen as a sole epithelial component in the present case, is uncommon. None of the reported cases of undifferentiated carcinoma of the gallbladder have heterologous mesenchymal elements. Focal chondrosarcoma in this case was evident although after diligent search. An extensive sampling and careful search for various epithelial and sarcomatous elements are mandatory for accurate diagnosis, in an attempt to decide appropriate clinical management and to try and improve the prognosis.
